# Rectal artesunate: lives not saved

**DOI:** 10.1093/trstmh/trae036

**Published:** 2024-05-25

**Authors:** N J White, T Peto, J A Watson

**Affiliations:** Mahidol Oxford Tropical Medicine Research Unit, Faculty of Tropical Medicine, Mahidol University, Bangkok 10400, Thailand; Centre for Tropical Medicine and Global Health, Nuffield Department of Medicine, University of Oxford, New Richards Building, Old Road Campus, Roosevelt Drive, Oxford, OX3 7LG, UK; Mahidol Oxford Tropical Medicine Research Unit, Faculty of Tropical Medicine, Mahidol University, Bangkok 10400, Thailand; Centre for Tropical Medicine and Global Health, Nuffield Department of Medicine, University of Oxford, New Richards Building, Old Road Campus, Roosevelt Drive, Oxford, OX3 7LG, UK; Centre for Tropical Medicine and Global Health, Nuffield Department of Medicine, University of Oxford, New Richards Building, Old Road Campus, Roosevelt Drive, Oxford, OX3 7LG, UK; Oxford University Clinical Research Unit, Hospital for Tropical Diseases, 764 Vo Van Kiet, District 5, Ho Chi Minh City, Vietnam

Each day, severe malaria is still estimated to kill approximately 2000 people, most of whom are African children. Early access to effective antimalarial treatment is the key to preventing death. The treated mortality of severe malaria in children should be <10%, whereas the untreated mortality is close to 100%. Severe malaria commonly occurs in places that are remote and poorly served by health services and where delays in receiving effective antimalarial treatment often have fatal consequences. Artesunate is the best treatment of severe malaria. Rectal artesunate (RAS) provides a simple, safe and convenient method of treating suspected severe malaria in the community, and thereby avoiding potentially lethal delays in reaching the health centre or hospital and starting parenteral antimalarial drug therapy.^1^ In January 2022, the WHO suddenly and unexpectedly advised a moratorium on the deployment of RAS.^2^ Fifteen months later, after much debate, the WHO repositioned their stance on RAS and issued a cautious recommendation that provision of an ‘effective continuum of care is a necessary prerequisite for the introduction of RAS’.^3^ However, the sluggish rise in RAS deployment before the January 2022 moratorium has now turned into a sharp decline (Figure 1). We argue that this has cost lives.

**Figure 1.**
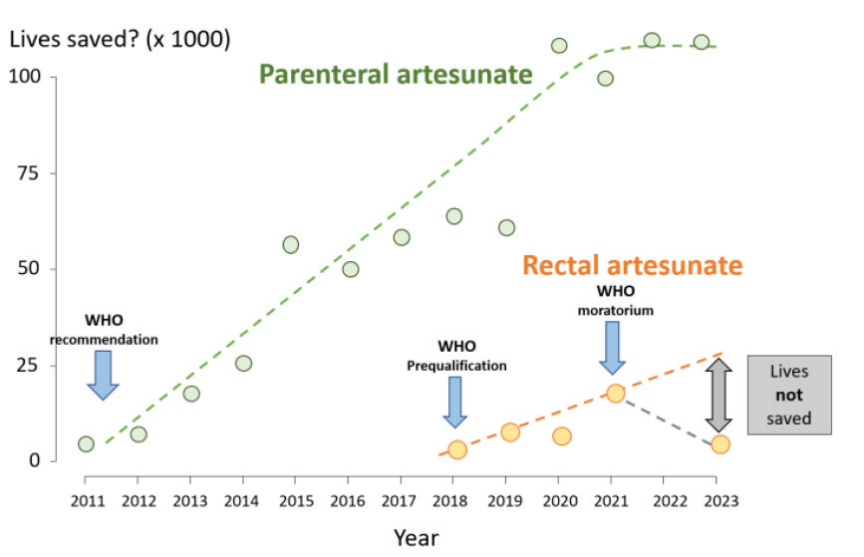
Estimates of severe malaria lives saved based on global procurement information under the following assumptions: (a) for parenteral artesunate, the number of patients needed to treat to save one life was 42,^3^ and the average dose used was five vials, of which one-half was used to treat suspected severe malaria; and (b) for rectal artesunate, the context of use was similar to that in the large randomised trial,^5^ the average number of RASs used was 1.5 (i.e. an equal split in ages between <3 y [one suppository] and >3 y [two suppositories) and the number of patients needed to treat to save one life was 241.

## Artesunate saves lives

Artesunate is the treatment of choice for severe malaria. The largest randomised controlled trial in patients hospitalised with severe malaria was conducted in African children (AQUAMAT: N=5425). It compared parenteral artesunate with quinine, the previous first-line treatment, and it showed a 23% reduction in mortality in favour of artesunate: 10.9% (quinine) vs 8.5% (artesunate).^4^ Compared with parenteral quinine, the average number of children needed to treat (NNT) to save one life in this trial was 42.

Ready access to healthcare is usually poor across the tropical malaria-endemic regions. To address the potentially lethal delays in receiving effective antimalarial treatment in these remote settings, a rectal formulation of artesunate (i.e. RAS) was developed^4^ that could be given by community health workers as a prereferral treatment for suspected severe malaria. RAS was evaluated in a very large community-based, double-blind, placebo-controlled randomised study (Study 13; N = 12 068 participants were evaluated).^5^ The results were first reported in 2009. Mortality was 3% in the placebo group vs 2.5% in those randomised to RAS. Overall, this difference was not significant, but this study reflected the reality of treating sick children in remote areas. Many of the randomised patients suspected of having severe malaria by the community health workers and entered into the randomised study did not have malaria. Severe malaria is overdiagnosed in African children, even in hospital research centres.^6^ But in those patients in Study 13 who did have malaria, and whose referral was delayed by >6 h (i.e. the circumstances in which RAS was expected to provide benefit), mortality was reduced by one-half (1.9% vs 3.8%) in the RAS recipients.^5^ However, it is the overall results from Study 13 that reflect the usual context of RAS use (i.e. that many of the children who are diagnosed as having severe malaria in the community or in health centres and who receive RAS will not have severe malaria). The overall figures from Study 13 suggest an average approximate NNT to save one life of 241. RAS is rapidly effective, safe and well tolerated. There have been concerns that RAS use could select for artemisinin resistance, but under most conditions single-dose administration cannot do this,^7^ so there is likely to be little or no selection pressure on artemisinin resistance from RAS use.

## The WHO moratorium on RAS

Since 2009, the development and deployment of RAS has gone very slowly. WHO prequalification was only obtained in 2018. This allowed purchase of RAS by international donors such as United Nations agencies and the Global Fund to fight HIV, TB and malaria. Purchasing and deployment of RAS then increased slowly, reaching a peak of 3.9 million rectal capsules in 2021 (Hans Rietveld. MMV: personal communication). Then, suddenly, in January 2022, after internal review of a then unpublished observational study (the CARAMAL study), the WHO Malaria Policy Advisory Group (MPAG) recommended a moratorium on further deployment of RAS.^2^ CARAMAL had reported that RAS deployment was associated with a fourfold increase in childhood mortality in Nigeria. Since then, the many problems with the CARAMAL study have been identified and discussed extensively. The WHO's own commissioned external review later concluded that a causal relationship between RAS deployment and increased mortality in the CARAMAL study ‘was not supported by the study design and data collection’.^3^ But this episode has had severe and long-lasting ramifications.

## WHO repositioning

In April 2023, following the report of the external commissioned review that cast doubt on the interpretation of the CARAMAL study, the WHO MPAG repositioned its stance on RAS. The WHO MPAG now concluded that the results of the CARAMAL observational study emphasise that ‘provision of an effective continuum of care is a necessary *prerequisite* for the introduction of RAS’.^3^ We argued that this recommended restriction of RAS deployment to settings that could provide ‘an effective continuum of care’ was harmful: that RAS was developed specifically for situations where health structures were weak and there was not ‘an effective continuum of care’, and that the greatest benefit from RAS would be in exactly those resource-limited settings.^1^ The WHO also announced that a field manual was now under preparation ‘which clearly outlines the conditions under which the introduction of RAS can be effective’.^3^ The manual ‘*Pre-referral treatment with rectal artesunate of children with suspected severe malaria: a field guide*’ was published by the WHO in November 2023.^8^ It states, somewhat ambiguously, that there are ‘Minimal essential requirements for RAS to be effective’ and that ‘To be effective, RAS requires minimal health system elements’, but it also states that ‘Countries should not base a decision to use on these requirements but rather work to strengthen the health system for optimal RAS implementation’. It then lists the 32 ‘*Essential minimum considerations for RAS deployment*’.

**Selection from the WHO Field Guide's 32 Essential Minimum Considerations for RAS deployment.** ^8^

• Manage health care system comprehensively, with all relevant actors, including the private sector

• Ensure availability of diagnostics and other drugs required for case management

• Patients should be referred directly to a facility that can offer emergency care and adequate post-referral treatments (in the case of severe malaria usually secondary or tertiary facilities)

• Support for impoverished communities, e.g. subsidised emergency transport system or social health insurance schemes to support increased completion of referral

• Affordable referral support (e.g. subsidized transport)

These are all laudable and important goals, but potential implementers in low-resource settings with weak health systems (i.e. the very places where RAS can save the most lives) may consider this large number of ‘essential minimum requirements’ unachievable, at least in the short term, and therefore defer or delay the deployment of RAS.

## The fatal consequences of the WHO moratorium

What has happened in the 2 ys since the WHO moratorium? The negative effect of these varying restrictions and cautions on RAS deployment has been substantial. The previous slow increase in RAS deployment, which should have accelerated beyond 2021, has now become a marked decline (Figure 1). Based on the estimated NNT to save one life with RAS and the difference between the projected and actual deployment, it may be concluded that thousands of childhood malaria deaths have not been prevented. Using the relatively conservative NNT estimate to save one life of 241, and assuming each suppository was used, and with an average of 1.5 suppositories per administration (i.e. an equal usage split between <3 y [one suppository] and >3 y of age [two suppositories]), suggests that >10 000 children’s lives were not saved in 2022 and 2023. Although this estimate is approximate, it is unlikely to be far from the unfortunate truth.

These estimates are imprecise, and they may be too low. The magnitude of the life-saving benefit of RAS in operational use, and thus the estimated NNT to save one life, was derived from the only large randomised trial. The overall result from Study 13 was not statistically significant because of the dilution by patients who did not have malaria, but there is very strong evidence that artesunate does save lives in the treatment of severe malaria.^1,4^ Importantly, the relatively low mortality in Study 13^5^ reflects the unusually good settings, training and referral systems created specifically to support that trial, and so it probably underestimates the overall benefit when RAS is deployed in remote, poorly served and difficult to access malarious areas. We have argued that the true benefits of RAS are likely to be greatest where health systems are weakest and access is poorest.^1^

### Conclusions

Severe malaria deaths can be prevented. RAS should be deployed much more widely. The adverse consequences of the January 2022 WHO moratorium on RAS have been serious and far reaching, and they have not been adequately reversed.

## Supplementary Material

trae036_Supplemental_Files

## Data Availability

The data underlying this article are provided in the article.
