# Supplementary material for: Rectal artesunate: lives not saved
Source: Trans R Soc Trop Med Hyg. 2024 May 25;118(10):639–41. doi: 10.1093/trstmh/trae036 (PMC11443335; doi:10.1093/trstmh/trae036)
Supplement: trae036_Supplemental_Files [file trae036_supplemental_files.zip › trae036 supplementary table.docx]

**trae036 - Rectal artesunate: lives not saved**

*Supplementary Table* : Annual procurement of parenteral artesunate vials (millions) and rectal artesunate suppositories/capsules (millions)

| Year | Artesunate injection vials | Rectal artesunate suppositories/capsules |
| --- | --- | --- |
| 2011 | 1.6 |  |
| 2012 | 3.2 |  |
| 2013 | 9.0 |  |
| 2014 | 12.1 |  |
| 2015 | 27.0 |  |
| 2016 | 24.6 |  |
| 2017 | 28.5 |  |
| 2018 | 30.0 | 0.7 |
| 2019 | 28.0 | 1.5 |
| 2020 | 49.9 | 1.2 |
| 2021 | 47.3 | 3.9 |
| 2022 | 51.6 | 1.7 |
| 2023 | 49.9 | 1.1 |
