# Supplementary material for: Rectal artesunate: lives not saved
Source: Trans R Soc Trop Med Hyg. 2024 May 25;118(10):639–41. doi: 10.1093/trstmh/trae036 (PMC11443335; doi:10.1093/trstmh/trae036)
Supplement: trae036_Supplemental_Files [file trae036_supplemental_files.zip › trae036 supplementary table.pdf]

trae036 - Rectal artesunate: lives not saved

Supplementary Table : Annual procurement of parenteral artesunate vials (millions) and rectal artesunate suppositories/capsules (millions)

Year	Artesunate	Rectal artesunate
2011	1.6	
2012	3.2	
2013	9.0	
2014	12.1	
2015	27.0	
2016	24.6	
2017	28.5	
2018	30.0	0.7
2019	28.0	1.5
2020	49.9	1.2
2021	47.3	3.9
2022	51.6	1.7
2023	49.9	1.1
